# Molecular Dynamics Simulation of Zeolite-Assisted Pyrolysis of Polystyrene: Material Selection and Mechanistic Insights

**DOI:** 10.1021/acs.iecr.4c03488

**Published:** 2024-12-04

**Authors:** Shuangxiu Max Ma, Changlong Zou, Bhavik R. Bakshi, Li-Chiang Lin

**Affiliations:** a William G. Lowrie Department of Chemical and Biomolecular Engineering, The Ohio State University, Columbus, Ohio 43210, United States; b School of Sustainability, Arizona State University, Tempe, Arizona 85281, United States; c School for Engineering of Matter, Transport and Energy, Arizona State University, Tempe, Arizona 85281, United States; d School of Complex Adaptive Systems, Arizona State University, Tempe, Arizona 85281, United States; e Department of Chemical Engineering, National Taiwan University, Taipei 10617, Taiwan

## Abstract

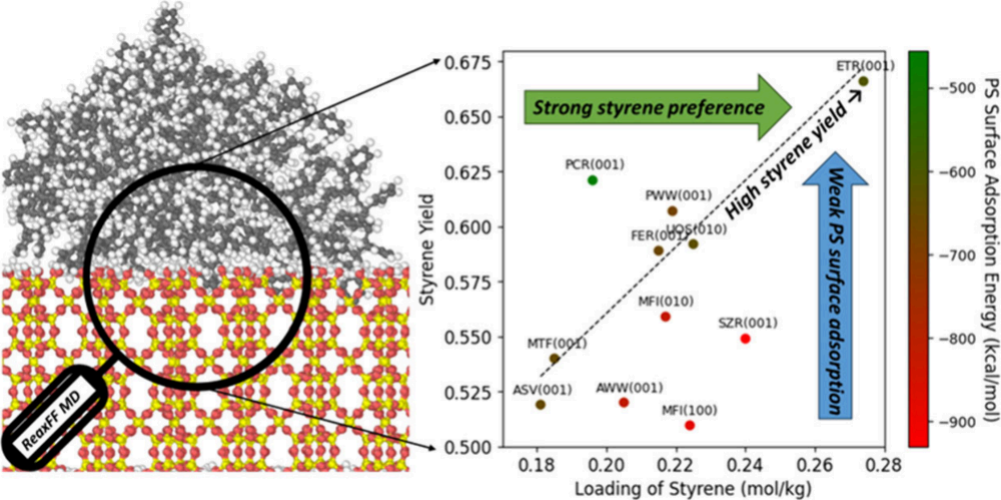

Polystyrene (PS) presents a significant environmental challenge due to its durability and resistance to degradation. A major issue in addressing this challenge is optimizing the pyrolysis process to selectively convert PS into valuable products, such as styrene, while minimizing unwanted byproducts. Existing studies on PS pyrolysis have primarily focused on general reaction yields and kinetics, with limited molecular-level insight into how zeolites can enhance product selectivity. This study addresses these gaps by investigating zeolite-assisted PS pyrolysis using a combination of reactive molecular dynamics (MD) simulations and Monte Carlo (MC) simulations. We specifically assess how zeolite structure and adsorption properties influence the pyrolysis product distribution, identifying optimal zeolites that enhance styrene yield. Our findings reveal that PS degradation occurs primarily through a chain-breaking mechanism without long-chain re-formation and that zeolites can significantly improve the selectivity and efficiency of the pyrolysis process by selectively adsorbing styrene. This work highlights the potential of zeolite-enhanced pyrolysis as a pathway for sustainable plastic recycling, advancing chemical recycling technologies to tackle plastic waste.

## 1 Introduction

The growing accumulation of plastic waste, particularly polystyrene (PS), poses significant environmental challenges^1,2^ owing to its durability and resistance to degradation. However, the global demand on plastic products continues to escalate, driven by their widespread applications in packaging, construction, and electronics. The imperative need for sustainable waste management strategies is therefore evident.^3,4^ Chemical recycling techniques, such as pyrolysis, have emerged as promising avenues for converting plastic waste into valuable hydrocarbon products, offering a potential pathway to mitigate environmental pollution and transition toward a circular economy.^5^ Pyrolysis, a process characterized by the thermal decomposition of polymers in the absence of oxygen, facilitates the transformation of plastic waste into a range of smaller hydrocarbon molecules, liquids, and gases.^6,7^ The versatility and environmental efficacy of pyrolysis hinge on its ability to accommodate various types of plastics and its potential for lower environmental impact compared to conventional waste disposal methods.^8,9^ However, enhancing pyrolysis performance, particularly in optimizing product distribution and reducing unwanted byproducts, remains a crucial area of research.

One promising approach to achieve this optimization of products is through the use of zeolites as adsorbents in the pyrolysis process. Zeolites, with their well-defined pore structures, can influence product distribution by enhancing the selectivity and efficiency of the process. This consequently allows for the targeted production of specific hydrocarbons while minimizing unwanted byproducts, thereby improving the overall effectiveness of pyrolysis. Miandad et al.^10^ and Rehan et al.^11^ showed that natural zeolite can improve the yield of liquid products that contain styrene and ethylbenzene, Ojha et al.^12^ demonstrated that the type of zeolite correlates with the yield of key products; yields of styrene increased, whereas those of benzene and indene derivatives decreased with the presence of beta zeolite. This suggests that zeolites’ affinity for styrene may lead to higher yields of styrene. Furthermore, Puente et al.^13^ found that styrene would be mainly produced from the thermal cracking of the polymer as the first step, and introducing zeolite to the PS pyrolysis can influence the equilibrium of the reaction and further improve the yield of styrene.

However, it is also observed that the yield of styrene does not always increase with zeolites’ affinity for styrene, suggesting the importance of strategic structural selections. As Valizadeh et al.^14^ pointed out, despite the promising results for applying zeolites in PS pyrolysis, there are still challenges in fully understanding how zeolites affect the efficiency of thermal pyrolysis and their affinity for targeted products. Addressing these issues is essential to develop more robust design rules and optimize the pyrolysis process. The lack of detailed molecular-level studies and systematic comparisons across different zeolites also challenges the objective assessment of zeolites’ roles in pyrolysis applications.

Understanding the pyrolysis process and complex interactions between the polymer and the zeolite surface remains a formidable challenge. Experimentally monitoring these intricate reaction pathways and interfaces at the molecular level is exceptionally difficult. To this end, computational simulations emerge as crucial tools that are capable of providing atomistic insights into the dynamics of plastic pyrolysis. At the forefront of these innovations are reactive molecular dynamics (MD) simulations, which utilize reactive force fields (ReaxFF) to describe the formation and dissociation of chemical bonds. This approach allows for a detailed atomic-level investigation of chemical reactions and material behaviors under various conditions, offering a deeper understanding of the mechanisms driving plastic pyrolysis.^15^ For example, Lu et al.^16^ studied the pyrolysis mechanism of polyimide, and Hu et al.^17^ investigated the thermal decomposition initiation mechanisms and kinetics of poly(α-methylstyrene) under isothermal conditions and noticed that the activation energy varies with the degree of conversion during the thermal decomposition processes. Early stage copyrolysis of typical plastic waste, including polyethylene (PE), polypropylene (PP), and polystyrene (PS), was also studied by He and Chen^18^ with a focus on improving the yields of tar and oil under higher temperatures. Besides, reactive MD simulations offer a profound understanding of how various factors (such as temperature, pressure, and catalysts) that influence the product distribution and efficiency of the pyrolysis process.^19^ However, for PS pyrolysis, previous theoretical research mainly focused on the calculation of kinetic constant^20^ or yield distribution of cracking process under complex situations such as external shock compression,^21^ extremely high temperature with low density,^22^ and copyrolysis with hydrogen donor,^23^ research specifically aimed at unraveling the detailed mechanism of PS pyrolysis remains lacking.

On the other hand, understanding the adsorption of targeted products in zeolite is also of vital importance, and Monte Carlo (MC) techniques have been proven to be an efficient method for capturing the adsorption properties of materials. For instance, Wang et al.^24^ utilized MC simulations to study metal–organic frameworks (MOFs) for harvesting atmospheric water and shed light on the selection of better adsorbents. Yang et al.^25^ also studied the adsorption of CO_2_ by amine-functionalized MOFs, employing the so-called grand canonical Monte Carlo (GCMC), and provided further insights into capacity degradation and how humidity influences the utilization rates of amines. Considering a large number of zeolites can be utilized as adsorbents, screening for appropriate structures is essential. Henry’s constants (KH) and the geometric properties of adsorbates can serve as useful indicators for selecting proper structures. This approach helps narrow down the range of zeolites of interest, making the selection process more efficient. This computer-aided materials selection has been successfully applied to a variety of applications, such as the separation of CO_2_ and N_2_ from wet flue gas,^26^ trace PH_3_ capture from hydrogen,^27^ propane capture from the air, and natural gas mixtures.^28^ Sholl et al.^29^ also suggested general rules for using the material with KH larger than 10^–3^ mol/kg/Pa for the selection process of targeted adsorbents, which is also adopted in this current study.

Overall, motivated by the need to understand PS pyrolysis and the effects of zeolites on a detailed molecular level and identify optimal zeolites for enhanced yields, this study combines reactive MD and MC simulations to investigate how adsorption can help increase the yield of styrene. While previous studies on PS pyrolysis have largely focused on kinetic constants, activation energies, or yield distributions under extreme conditions, the specific molecular interactions between PS and zeolites, especially regarding product selectivity and adsorption properties, remain underexplored. This study addresses these gaps by investigating how zeolite structure and adsorption characteristics influence pyrolysis product distribution and identifying zeolites that can preferentially adsorb styrene, thereby enhancing its yield. By employing these advanced simulation techniques, deeper insights into the interactions between PS pyrolysis products and various zeolites can also be achieved, ultimately identifying and understanding effective zeolite structures for maximizing styrene production. The novel contributions of this work include a methodical approach to screening zeolite candidates for styrene affinity and adsorption capacity without the need for exhaustive ReaxFF MD simulations across the entire zeolite database. By combining MD simulations to capture the dynamic interactions and MC simulations to assess adsorption behavior, this study offers a unique framework for evaluating zeolites in the context of plastic pyrolysis. Specifically, we (1) analyze the pyrolysis process of pure PS to establish the primary reaction mechanisms and main species involved, (2) systematically screen zeolite structures based on their affinity for styrene, and (3) evaluate the impact of PS-zeolite interfaces on product distribution using ReaxFF MD simulations. The outcomes of this work demonstrate efficient identification of high-performing zeolite structures without the need for exhaustive ReaxFF MD simulations for the entire zeolite database. By achieving higher yields of valuable products like styrene, this research holds significant environmental and economic implications. Efficiently converting PS waste into valuable hydrocarbons via zeolite-assisted pyrolysis reduces the need for landfill disposal and contributes to a circular economy, where waste materials are reintegrated into production cycles. Enhanced product selectivity also minimizes unwanted byproducts, leading to cleaner processes with lower waste output. Furthermore, the identification of optimal zeolite structures can make PS pyrolysis more economically viable by reducing energy consumption and improving reaction efficiency.

## 2 Computational Details

In this section, the detailed rules for selecting zeolite candidates will be first given and followed by the details of MC simulations are provided. Then the modeling of the pure PS and PS-zeolite systems is described to provide a graphic understanding of the systems in this study. Finally, the ReaxFF parameters used in the study are then provided.

### 2.1 Zeolite Selection Process

Since it is computationally intensive to carry out ReaxFF MD simulations for the PS pyrolysis assisted with approximately 240 different zeolite structures from the International Zeolite Association (IZA) database,^30^ it is important to reduce the number of materials of interest. In this study, we employ a multistep selection process.

**Figure 1:**
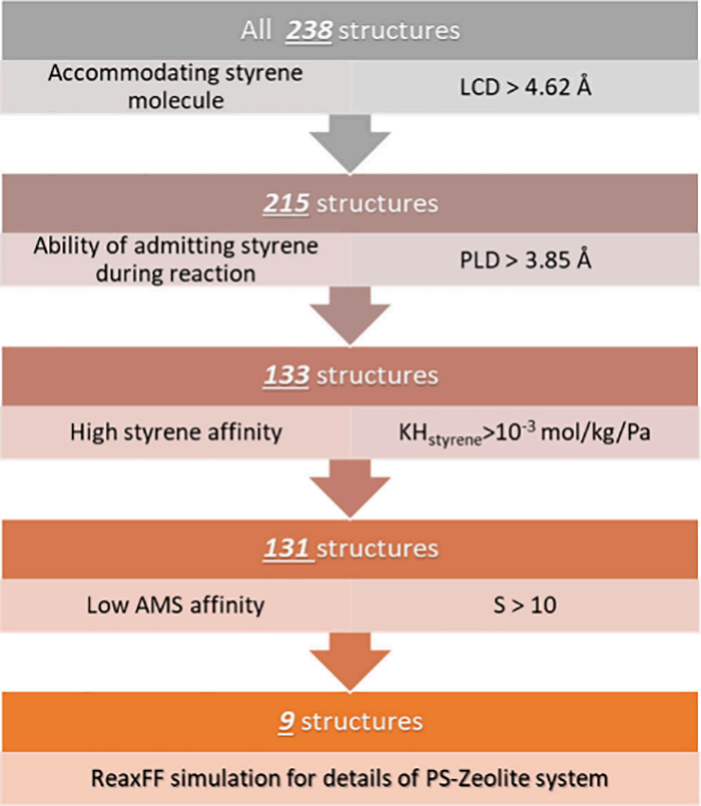
Schematic illustration of the four steps employed to identify nine potential candidates from 238 zeolites in the IZA database.

As depicted in Figure 1, zeolites with the largest cavity diameter (LCD) of greater than 4.62 Å are first selected, as they can accommodate styrene molecules. Refinement is then achieved by evaluating the ability of the zeolites with a PLD greater than 3.85 Å, indicating a sufficient pore size to allow the diffusion of styrene into the structure. To emphasize the capability of the zeolite to attract styrene, KH for styrene and α-methylstyrene (AMS) are used as material selection criteria and are computed via the Widom particle insertion method^31^ implemented in the RASPA package^32^ with the force fields discussed in the following section. We use the constraints of KH values as suggested in the earlier work by Sunrr et al.^33^ and Sholl et al.:^29^ Only those zeolites with a KH_styrene_ greater than 10^–3^ mol/kg/Pa are considered, leading to a total of 131 structures. Additionally, it is important to depress the adsorption of AMS, a major side product of PS pyrolysis. The selectivity (S) based on the KH is defined as KH_styrene_/KH_AMS_. Subsequently, zeolites with S > 10 are selected, further narrowing the candidates to nine zeolites. Additionally, five other unique cases are incorporated for systematic comparison: the MFI framework with three different surfaces, UOZ as an example with a PLD smaller than the standard selection, and AEL noted for its high AMS affinity.

### 2.2 Adsorption Properties

The Widom particle insertion implemented in the RASPA package^32^ is employed in all KH calculations at 300 K, with the rigid assumption for both molecules and frameworks. The force field developed by Emami et al.^34^ is adopted for zeolites, and the OPLS-AA^35^ force field is utilized for both styrene and AMS. The Lennard-Jones (L-J) potentials are truncated and shifted to zero at 12 Å, while the long-range interactions are computed using the Ewald summation method. It is worth mentioning that a prior investigation conducted by Zou and Lin demonstrated that the utilized force field in this study could reasonably describe the adsorption phenomenon for zeolites.^36^ GCMC simulations (also implemented in the RASPA package^32^) are also carried out under 1800 K to compute the adsorption isotherms of styrene/AMS mixtures in zeolites in order to further understand the performance of selected zeolites under reaction conditions. While there may exist a broad range of styrene/AMS ratios during the pyrolysis reaction, an equal molar condition (i.e., 1:1 styrene:AMS) is used in this study for simplicity. It is noted that while other products from PS pyrolysis could also serve as competing adsorbates with styrene and AMS in the zeolite-assisted PS pyrolysis, this study primarily focuses on styrene and AMS since the yields of those side products are very low. Configurations of adsorbates are sampled through millions of translations, rotation, reinsertion, swap, and identity change moves with a ratio of 1:1:1:2:2 while the same force field is applied. It should also be noted that it is exceptionally challenging or probably not possible to ensure that the adopted generic force field can accurately capture the adsorption properties of all studied adsorbents. However, it is anticipated that the trend or the relative performance of different adsorbents may be decently described.

### 2.3 Molecular Dynamics (MD) Simulations and PS-Zeolites Models

**Figure 2:**
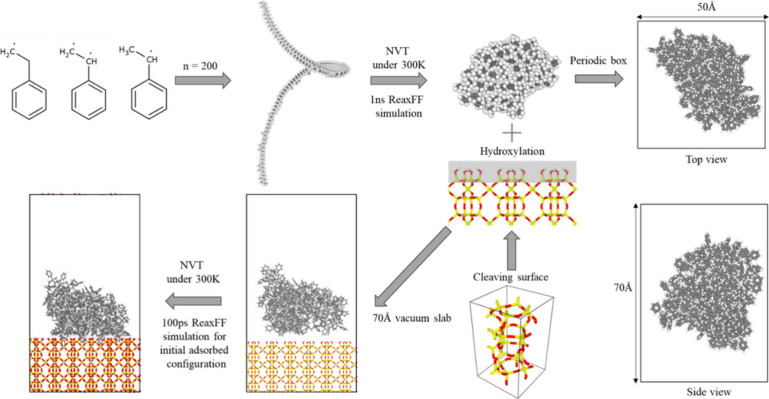
Illustration of the procedure to build the simulation systems for pure and zeolite-assisted PS pyrolysis. For the former, a long PS chain consists of 200 monomers (i.e., *n* = 200), and the molecule is placed in a large periodic domain. For the latter, a zeolite layer is further incorporated, followed by relaxation under the canonical ensemble (NVT) at 300 K. The structure of ASV is used in this figure for illustrative purposes.

Figure 2 schematically illustrates the systematic procedure to construct the simulation system for PS pyrolysis and that assisted by zeolites (i.e., zeolite-assisted PS pyrolysis). The process begins with the generation of a long PS chain by the Moltemplate package,^37^ where a repeating monomer unit is polymerized to achieve the desired chain length (e.g., 200 monomers, denoted as n = 200). All the simulations of pyrolysis are performed with the ReaxFF, which is designed for complex reactive systems. By accurately capturing the reactive behavior of these complex systems, ReaxFF facilitates a detailed analysis of both the pure PS pyrolysis and the zeolite-assisted pyrolysis process. The long PS chain is first relaxed under the NVT ensemble—constant number of particles (N), volume (V), and temperature (T)**—**to reach a packed polymer structure. This PS polymer is then positioned within a large periodic domain, allowing for the simulation of a realistic polymer-zeolite structure.

We note that the volume of each studied zeolite structure is meticulously adjusted to ensure consistency across simulations, and the surface is cleaved according to the direction of the pore limiting diameter (PLD) (see SI Table S1). This consideration is crucial for comparing different PS-zeolite systems. After introducing the zeolite into the simulation domain, following a methodology that has been successfully employed in our previous study,^38^ the system is relaxed again under the NVT ensemble to reach a state of equilibrium. The reactive MD simulations employing ReaxFF are then performed from the constructed PS-zeolite systems under an NVT ensemble with a Nosé–Hoover thermostat^39,40^ to simulate pyrolysis in the sealed reactor. The temperature of the systems is ramped up to the desired temperature of 1800 K at a heating rate of 100 K/ps, followed by holding at the target temperature for three nanoseconds. A time step of 0.1 fs and a temperature damping constant of 10 fs are used in all ReaxFF MD simulations. MD simulations are performed using the open-source LAMMPS code.^41^

### 2.4 Reactive Force Field (ReaxFF)

The simulation of pure PS pyrolysis and zeolite-assisted PS pyrolysis leverages the ReaxFF,^42^ a set of parameters with given mathematical expressions adopted to describe the bond order for the formation and dissociation of chemical bonds. The energy term is computed with bond energy (E_bond_), overcoordination energy (E_over_), undercoordination energy (E_under_), valence angle energy (E_val_), penalty energy (E_pen_), torsion energy (E_tors_), conjugation energy (E_conj_), as well as nonbond van der Waals (E_vdWaals_) and Coulomb (E_Coulomb_) interactions. The parameters utilized in our simulations are adopted directly from the study of Joshi and co-workers^43^ for systems containing elements of C, H, O, Si, and Al. These parameters are trained and validated against highly accurate density functional theory (DFT) calculations and have been successfully applied in numerous reactions with zeolites, such as the catalytic cracking of hexadecane on ZSM-5,^44^ the complex reaction of confined water in clay-zeolite systems,^45^ methane adsorption on the silica-kaolinite interface^46^ and the methanol-olefins transfer reaction catalyzed by zeolite.^47^

## 3 Results and Discussion

### 3.1 Selection of Potential Zeolites

**Figure 3:**
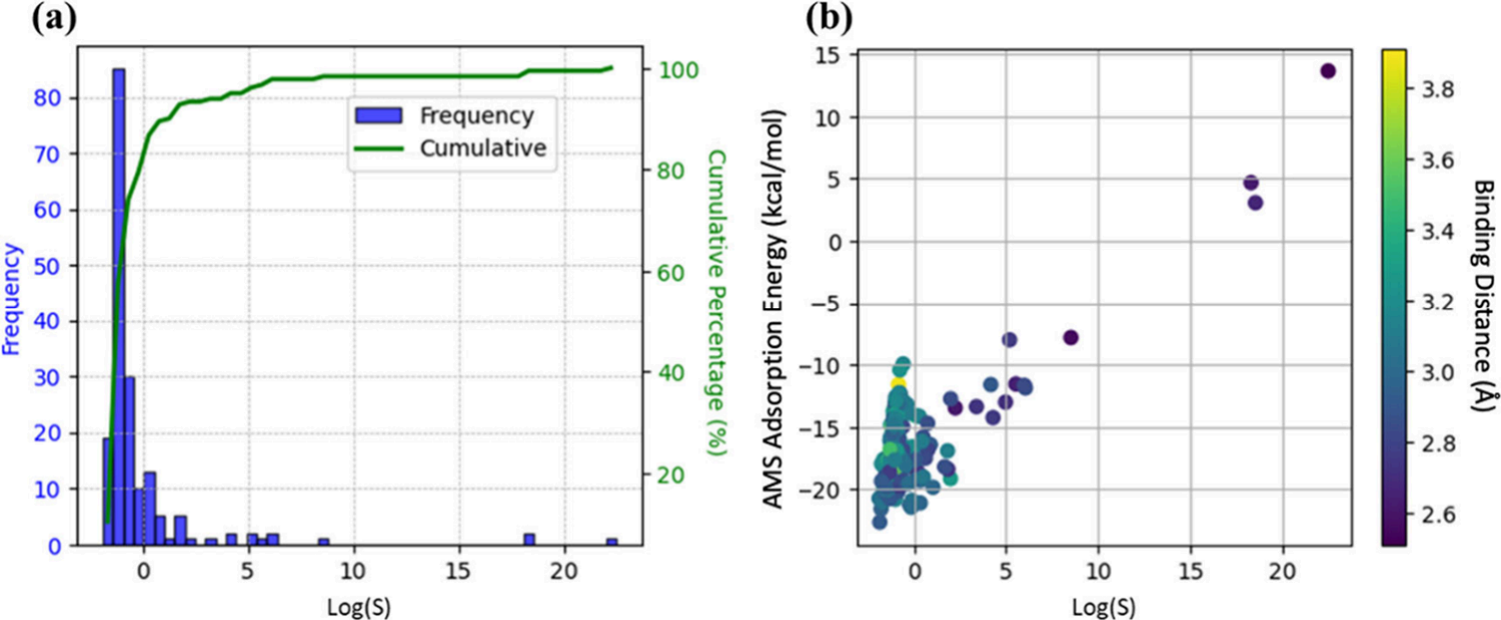
(a) Distribution of log(S) for all zeolites. (b) AMS adsorption enthalpy as a function of log(S) with each data point color-coded by the binding distance (i.e., the smallest Si–H distance between the adsorbate and zeolite framework).

Since simulating the PS pyrolysis with all the structures included in the IZA database (i.e., ∼240) with ReaxFF requires extremely high cost, a selection process is undertaken first. In this section, structures with proper LCD and PLD, as introduced in the methods section, are first selected, followed by computing their KH and S values. Figure 3a displays the cumulative distribution of the estimated log(S) for 131 selected zeolite candidates identified from the initial geometric selection. Zeolites with large log(S) values tend to have LCD values between 5 and 6 Å. However, the relationship is relatively weak, as shown in Figure S1a, because the LCD value represents only a local property of the structure and does not account for the detailed interactions between adsorbates and adsorbents. The results show that nine of all identified candidates can potentially offer a significant styrene affinity over AMS (with KH_styrene_ > 10^–3^ and log(S) > 1), which may serve as effective adsorbents in zeolite-assisted PS pyrolysis to improve the yield for styrene by preferentially removing styrene molecules from the gas phase into the zeolite and the low affinity of AMS is anticipated to effectively depress the generation of side product.

Additionally, the binding distance in Figure 3b is defined as the smallest distance between the H atoms in the AMS molecules and Si atoms in the zeolite framework, and it shows that the zeolites with both higher AMS adsorption energy and a smaller binding distance have a higher affinity of styrene over AMS. This finding is reasonable since the positive adsorption energy indicates the zeolites are AMS phobic, which will increase the log(S), and the compactness of the pore space can be seen as a vital feature of the difficulty of accommodating AMS. As shown in Figure S1b and the heatmap of feature correlation in Figure S2, the AMS adsorption energy and the binding distance are key to the styrene/AMS selectivity. Overall, with the consideration of the geometric properties and the adsorption properties, nine promising candidates are identified.

### 3.2 Yields and Loadings of Main Products

The yields of styrene and AMS using nine selected zeolites and five additional ones are analyzed. As noted above, for systematic comparison, five additional cases are incorporated aside from those nine potentially promising candidates: the MFI structure with three different surfaces, UOZ as an example with an excessively small PLD, and AEL with a high AMS affinity. These selections are made to offer a comprehensive understanding of how different zeolite characteristics affect the pyrolysis outcomes.

**Figure 4:**
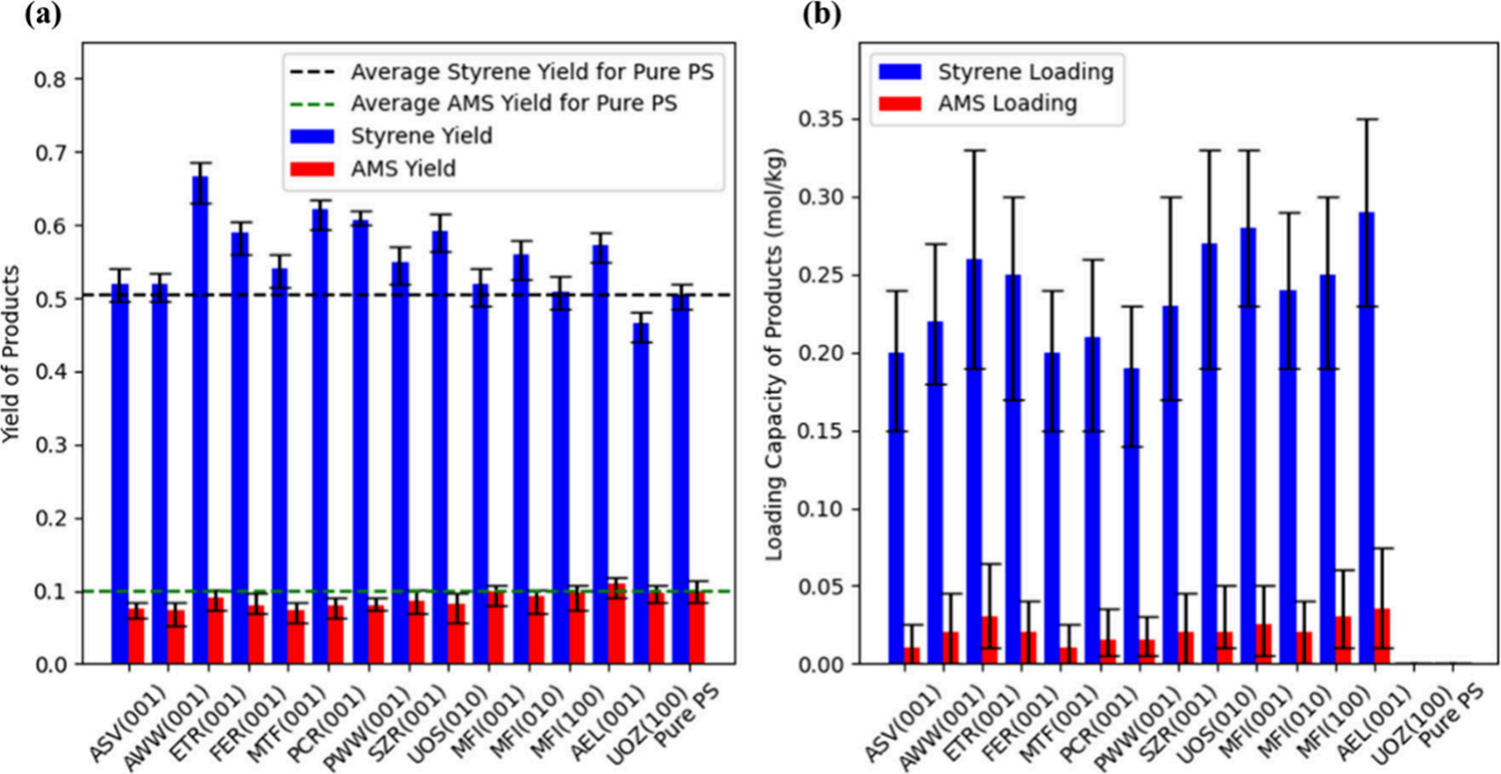
(a) Yield and (b) loading of styrene and AMS. The results of pure PS pyrolysis are also displayed for comparison. In (b), the uptake values for UOZ (100) and pure PS are shown as zero.

Figure 4a shows that zeolites capable of adsorbing styrene generally yield more styrene, and such enhancement can be by as much as 16% compared to pure pyrolysis. An exception is UOZ, a structure that cannot accommodate styrene molecules due to its small PLD. Additionally, the yields of AMS for most selected zeolites are also interestingly lower than those observed in pure PS pyrolysis, except for the AEL zeolite, which exhibits a higher AMS yield due to its high AMS affinity. Figure 4b further illustrates the loading of styrene and AMS in various zeolites. In correspondence to the results shown in Figure 4a, zeolites that are not conducive to adsorption, as indicated by lower loading values, tend to lower the yield for both styrene and AMS, highlighting the importance of selecting appropriate zeolite structures. This underscores the direct impact of adsorption properties on the yield of pyrolysis products. The enhanced yields of styrene in the presence of zeolites are closely linked to their adsorption properties, with zeolites that preferentially adsorb styrene over AMS significantly improving overall yield and efficiency.

### 3.3 Product Distribution of Zeolite-Assisted PS Pyrolysis

**Figure 5:**
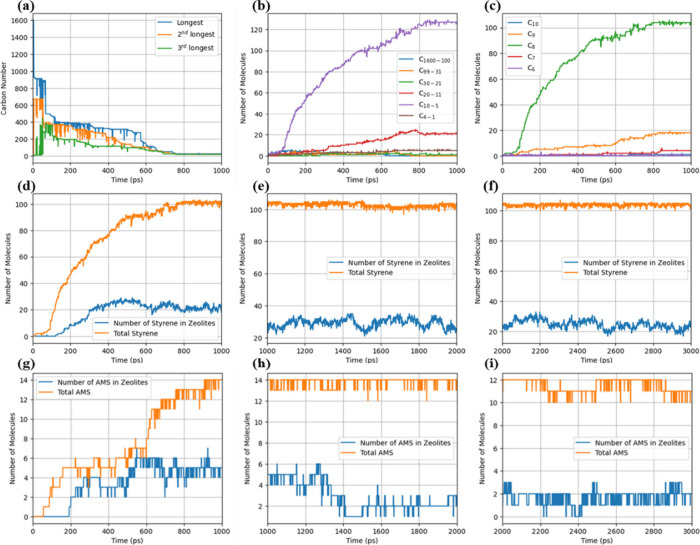
Time evolution of (a) the carbon number of 1st, 2nd, and 3rd longest chain species observed, (b) species classified with carbon numbers, and (c) species with carbon numbers from 6 to 10 at 1800 K for the PS-ASV system. (d–f) Time evolution of total styrene molecules and adsorbed styrene in zeolites, as well as (g–i) total AMS molecules and adsorbed AMS from 0 to 3 ns at 1800 K for PS-ASV system.

Reactive MD simulations were performed to investigate the pyrolysis of pure PS, focusing on identifying major species and understanding the underlying reaction mechanisms. Initially, we compared the observed products from PS pyrolysis in the simulations with experimental data (Table S2), validating ReaxFF’s capability to accurately capture the chemical composition during PS pyrolysis. The calculated activation energy for PS pyrolysis from MD simulations, 313.4 kJ/mol, closely aligns with the experimental kinetic value of 314.4 kJ/mol^48^ (Figure S3a), reinforcing the accuracy and reliability of ReaxFF in modeling pyrolysis systems. Furthermore, the detailed evolution and distribution of species observed in MD simulations are investigated. For simplicity, we focus on the PS-ASV system to illustrate the influence of zeolite on PS degradation with the detailed results of pure PS pyrolysis displayed in Figure S3. As shown in Figure 5a, the chain cracking of PS is still rapid, quickly releasing styrene. However, in the presence of zeolite, the initial degradation of the PS long chain is relatively slower compared to pure PS pyrolysis (Figure S3b), indicating that the interface formed by PS and the zeolite surface may negatively impact the pyrolysis rate.

Besides, similar to pure PS pyrolysis (Figure S3b and Figure S3c), short-chain species do not merge into longer chains, and styrene and AMS remain as the major species (Figure 5b and 5c), suggesting that the presence of zeolites does not alter the reaction mechanism or lead to new pathways for styrene production. Figure 5b also shows a lower quantity of C_1–4_ species (more details can be seen in Figure S4), implying that the zeolite does facilitate styrene adsorption and mitigate side reactions, preventing further decomposition of styrene by stabilizing reactive intermediates within its structure. As revealed in Figures 5d–f, styrene adsorption reaches a plateau within the first nanosecond and maintains this level up to 3 ns. In contrast, AMS shows an initial increase in adsorption, as shown in Figure 5g, followed by a decline in the simulations from 2 to 3 ns. AMS presence diminishes significantly in Figure 5h and 5i, further indicating preferential adsorption of styrene over AMS due to the zeolite’s high styrene affinity. Overall, ReaxFF MD simulations are performed on the selected zeolite systems, and when compared with pure PS pyrolysis, it is noted that the adsorption of styrene can increase the yield of styrene. A similar product distribution is observed, suggesting that the introduction of zeolite does not change the reaction mechanism.

### 3.4 Reaction Mechanism

**Figure 6:**
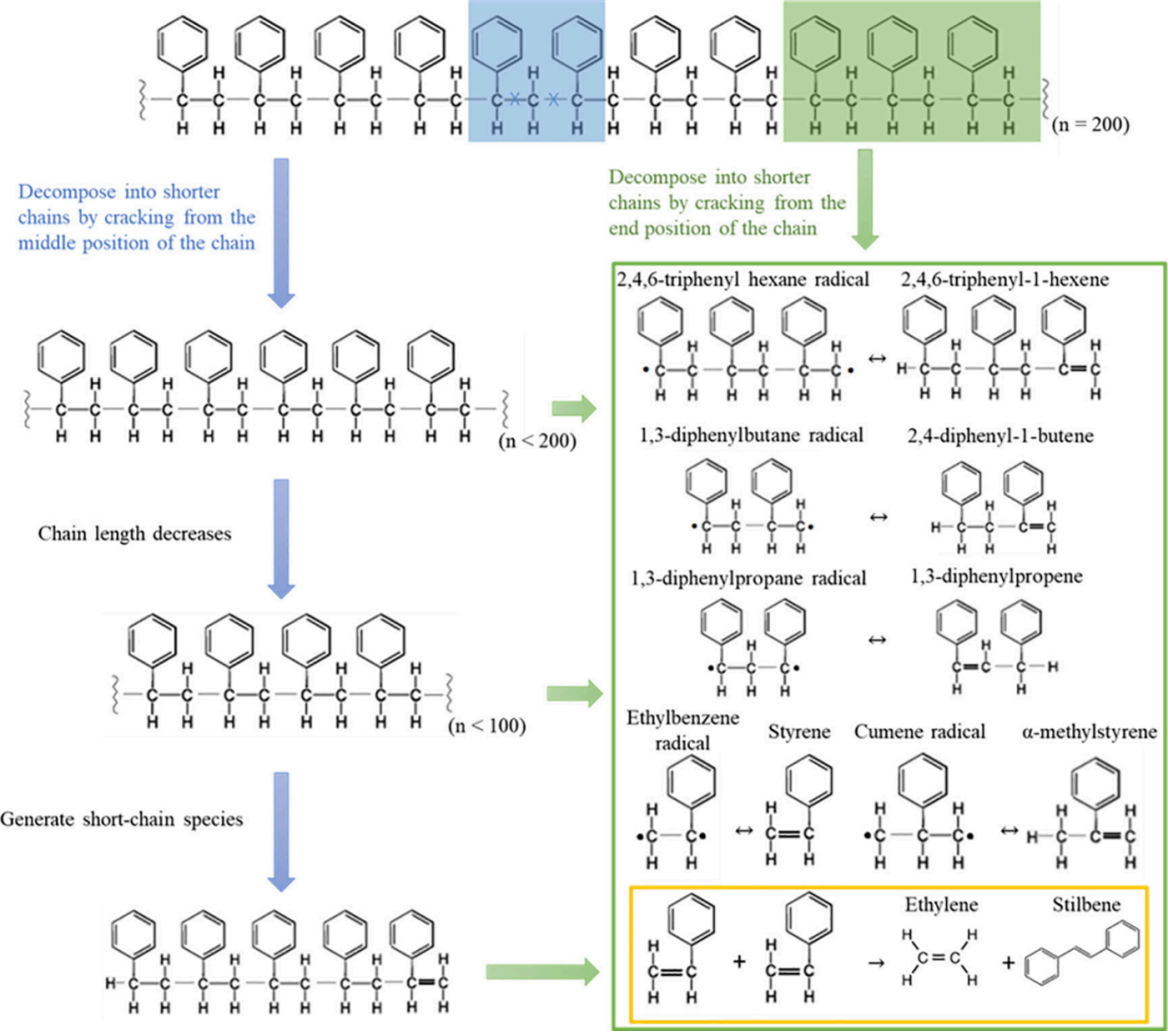
Schematic diagram of key reactions involved in PS pyrolysis observed from MD simulations. The blue and green arrows represent the reactions of long-chain species breaking from the middle or near the end of the long chain, respectively. The green frame displays the trimer, dimer, and monomer structures generated from end cleaving, while the yellow one shows the reaction pathway of generating ethylene.

As schematically depicted in Figure 6, a general cracking mechanism of PS pyrolysis is proposed. During the reaction, two primary pathways are observed: chain cracking from the midpoint (blue arrow) and cleavage at the end or near the end of chains (green arrow). This leads to the formation of trimer, dimer, and monomer radicals, which further decompose into phenyl-containing olefins, as shown in the green frame of Figure 6. In both pure PS pyrolysis and zeolite-assisted PS pyrolysis, chain length decreases rapidly at the onset of the reaction (Figure 5a and b), accompanied by the generation of long-chain species (C_1600–100_ and C_99–31_ in Figure 5b and Figure S3c). For each system, cleavage near the chain ends produces trimer and dimer radicals, with some radicals further decomposing into monomers, including styrene. Notably, the trimer and dimer radicals are released from long chains simultaneously, with the dimer potentially forming both from trimer and directly from long chains. Styrene molecules generated in this process will not undergo further direct cracking to produce C_2_ species or reform into long-chain species, which is consistent with the observation that chain growth halts in the later stages of pyrolysis.

It is observed that ethylene generation primarily results from olefin metathesis of styrene (yellow frame in Figure 6) and correlates with a lower observed C_6_ species, while stilbene quantities increase alongside ethylene production. The PS-zeolite interface, however, does modify the product distribution, even if the overall mechanism remains the same. Specifically, zeolites saturated with OH groups (and without Al) influence reaction dynamics by providing an adsorption site without directly altering the reaction chemistry. However, compared to pure PS pyrolysis, zeolites can depress the olefin metathesis reaction and lead to lower yields of dimers, trimers, and ethylene. Overall, this mechanism underscores that PS pyrolysis primarily proceeds through a chain-breaking process without long-chain reforming, with zeolite surfaces affecting only the distribution of specific light hydrocarbon products rather than the underlying reaction pathways.

### 3.5 Styrene/AMS Yield and Adsorption

**Figure 7:**
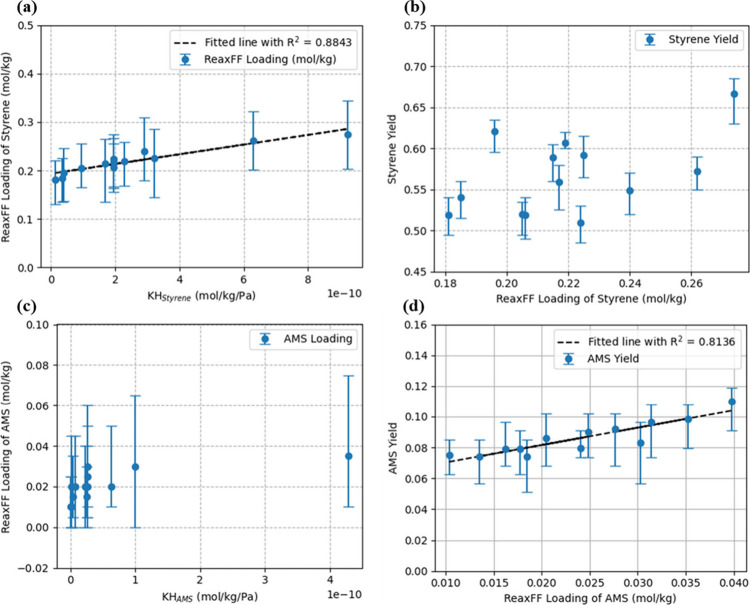
(a) ReaxFF loading of styrene vs KH and (b) styrene yield vs ReaxFF loading of styrene. (c) ReaxFF loading of AMS vs KH and (d) AMS yield vs ReaxFF loading of AMS.

To further quantify the relationship between yields and adsorption properties, Figure 7 displays the correlation between the loading capacity observed from ReaxFF MD simulations, Henry’s constant, and the product yield. Figure 7a clearly shows that the styrene loading from ReaxFF MD is positively correlated with KH. However, it is also interestingly noted in Figure 7a that styrene loading does not increase with KH_styrene_ in the same order of magnitude. This discrepancy arises because our system has deviated from the linear region where Henry’s law applies. Figure S5 reveals adsorption behaviors from GCMC simulations. Compared to the GCMC outcomes, saturation has not been achieved for these zeolites, but there is a noticeable deviation from the linear regime predicted by Henry’s law, except for UOZ, which shows zero loading. This explains the previous finding: although styrene loading positively correlates with KH_styrene_, it does not increase with KH_styrene_ in the same order of magnitude. Furthermore, the variation shown in Figure 7b also suggests factors other than loading capacity can significantly influence the styrene yield. This aspect will be further discussed in the following sections.

For AMS, similar to styrene, a positive relationship exists, and deviation is also observed in AMS loading relative to KH_AMS_, as shown in Figure 7c, indicating a similar divergence from Henry’s region. The results of saturation loading are shown in Figure S5a and S5b. From the comparison of the loading from ReaxFF MD simulations and GCMC simulations (Figure S5c and S5d), it is noted that AWW, SZR, and ASV have reached saturation, while other systems deviate from Henry’s region. Meanwhile, it is noted that even in AMS-phobic zeolites such as ASV, AMS can still be accommodated. This happens because styrene molecules that enter the zeolite after AMS can subsequently block AMS within the zeolite (see details in Figure S6). This blocking effect allows AMS to remain in the zeolite despite its initial aversion, resulting in a higher-than-predicted loading of AMS for AMS-phobic zeolites. Additionally, the low yield of AMS molecules from PS cracking inherently restricts the loading for zeolites such as ETR and AEL. This explains that for zeolites with higher AMS affinity, the loading does not grow with KH_AMS_ as expected, which leads to the selectivity calculated from ReaxFF MD differing from the selectivity calculated from KH (Figure S7). As for the yields, Figure 7d illustrates that the AMS yield is closely controlled by its ReaxFF loading, with a strong positive correlation indicated by an R^2^ value of 0.8136. As the ReaxFF loading of AMS increases, the yield of AMS correspondingly rises, demonstrating the significant impact of loading on the yield. This suggests that higher AMS loading of zeolites enhances the availability of AMS for reaction, directly influencing the yield. In summary, for both styrene and AMS, the loading capacity is positively related to KH. However, deviations from Henry’s region result in the loading capacity and KH not changing proportionally. Besides, it is noted that for styrene, other factors appear to influence the yield besides loading capacity.

### 3.6 Surface Adsorption of PS Polymer

**Figure 8:**
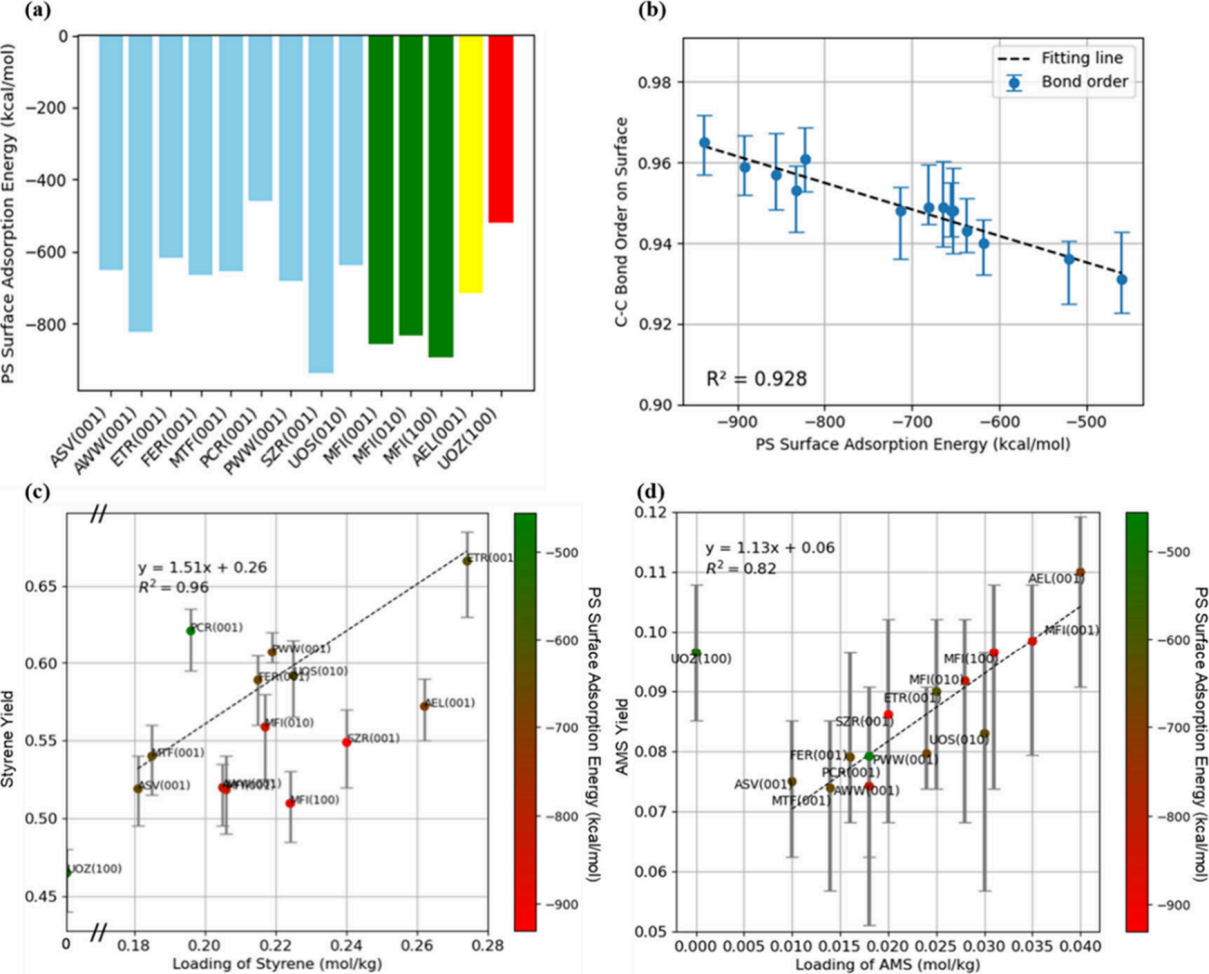
(a) The PS surface adsorption energies for 14 PS-zeolite systems. (b) C–C bond order near zeolite interface vs PS surface adsorption energy. (c) The styrene yield of the PS-zeolites system vs the loading of styrene, with each data point color-coded by the PS surface adsorption energy. The fitting line is plotted between the systems with similar PS surface adsorption energies (ASV(001), MTF(001), FER(001), PWW(001), and ETR(001)). (d) The AMS yield of the PS-zeolites system vs the loading of AMS, for which data points are color-coded by the PS surface adsorption energy.

From the previous section, it is found that the AMS yield is strongly correlated with loading capacity, while the styrene yield depends on additional factors beyond loading capacity. Herein, the effect of adsorption interactions between the PS polymer and the zeolite surface is further studied. As shown in Figure 8a, significant variations are interestingly observed in PS surface adsorption energies across the zeolite types. For example, MFI zeolites, shown in green, exhibit strong interactions with PS, with adsorption energies ranging from −832.44 to −892.08 kcal/mol. The AEL zeolite, highlighted in yellow, has a moderate adsorption energy of −713.1 kcal/mol. The UOZ zeolite, marked in red, shows notably weaker adsorption energy of −520.8 kcal/mol.

To quantify the influence of the PS surface adsorption, the relative density of the adsorbed PS is methodically determined as a function of distance (i.e., radius (R)) from the center of mass of the molecules). The computed density is then normalized by the corresponding value for pure PS to assess the relative density (i.e., equations S1, S2, and S3 in the SI). Figure S8a depicts the analytical relationship between surface adsorption energy and relative density. The results consistently demonstrate a clear trend: an increase in relative density correlates with stronger adsorption energy, suggesting a denser, more compact PS configuration upon stronger adsorption. This is also reflected in the greater number of carbon atoms on the surface, as shown in Figure S8b. Moreover, the bonding order is correspondingly found to increase (Figure 8b). Such a stronger bond consequently slows down the cracking of the PS polymer. It is noted that this compact PS configuration predominantly influences the initial stage of surface-mediated reactions, such as long-chain breaking and initial styrene production. As the reaction progresses and the chain length diminishes, the molecules move toward the vacuum slab.

Taking the PS surface adsorption energy into consideration, Figure 8c shows that the styrene yield is positively correlated with the loading of styrene for zeolites with similar PS surface adsorption energies (ASV(001), MTF(001), FER(001), PWW(001), and ETR(001)), with a correlation coefficient (R^2^) of as high as 0.96. Zeolites with weaker PS surface adsorption (e.g., PCR, in green) fall above this line, while those with stronger PS surface adsorption (in red) fall below it, indicating that stronger PS surface adsorption tends to lower the styrene yield. In contrast, Figure 8d shows that PS surface adsorption energy plays an insignificant role in AMS yield. This appears reasonable, given AMS breaks from shorter chains. Overall, this section elucidates how the PS surface adsorption determines the yield of pyrolysis products, highlighting the crucial role of the PS-zeolite interface in influencing product yields.

## 4 Conclusions

This study presents an integrated computational framework combining reactive MD simulations and MC calculations to investigate the zeolite-assisted PS pyrolysis. This approach enables a comprehensive understanding of the zeolite-assisted PS pyrolysis process, from the pyrolysis mechanism (i.e., including both the pure PS pyrolysis and that assisted with zeolites), polymer-zeolite interactions, to the final yield and its associated product distribution. After a multistep screening process, zeolite systems with high styrene affinity over AMS are identified for detailed ReaxFF MD simulations. It is observed that PS pyrolysis is mainly a chain-breaking process without long-chain reforming, and the decomposition mechanism of PS remains unchanged with the introduction of zeolites, emphasizing that the primary factor behind the enhanced yield is the adsorption characteristics of the zeolites. The results indicate that increased styrene affinity in zeolites indeed enhances the yield. However, the surface adsorption of PS polymers can lower the yield because the strengthened bonds within polymers near the interface are more difficult to break. Overall, integrating MC and ReaxFF MD simulations provides a robust approach to understanding the zeolite-assisted PS pyrolysis systems. This methodology elucidates fundamental mechanisms and revisits the design principles of zeolite-assisted PS pyrolysis. Future work could extend this methodology to other types of plastics to deepen our understanding of zeolite-assisted pyrolysis behavior and explore new zeolite materials to improve recycling processes.

## Supporting Information Available

Details of constructing PS-zeolite systems; styrene affinity and important features; feature correlations; comparison of main products observed in experiments and simulations; pyrolysis of pure PS; light species for PS-ASV system; comparison in loading capacity between GCMC and MD; comparison between loading and Henry’s constant; computational details of relative density distribution; Rrelative density distribution and carbon atom disrtibution (PDF)
