# Supplementary material for: Molecular Dynamics Simulation of Zeolite-Assisted Pyrolysis of Polystyrene: Material Selection and Mechanistic Insights
Source: Ind Eng Chem Res. 2024 Dec 4;63(50):21907–17. doi: 10.1021/acs.iecr.4c03488 (PMC11660221; doi:10.1021/acs.iecr.4c03488)
Supplement: Supplementary file 1 — ie4c03488_si_001.pdf [file ie4c03488_si_001.pdf]

Supporting Information

for

Molecular Dynamics Simulation of Zeolite-Assisted Pyrolysis of Polystyrene: Material Selection and Mechanistic Insights

Shuangxiu Max Ma[a], Changlong Zou[a], Bhavik R Bakshi[b,c,d,*], Li-Chiang Lin[a,e,*]

[a] *William G. Lowrie Department of Chemical and Biomolecular Engineering, The Ohio State University, Columbus, OH 43210, United States.*

[b] *School of Sustainability, Arizona State University, Tempe, AZ 85281, United States.*

[c] *School for Engineering of Matter, Transport and Energy, Arizona State University, Tempe, Arizona 85281, United States.*

[d] *School of Complex Adaptive Systems, Arizona State University, Tempe, AZ 85281, United States.*

[e] *Department of Chemical Engineering, National Taiwan University, Taipei 10617, Taiwan.*

[*] Corresponding authors: bhavik.bakshi@asu.edu, lclin@ntu.edu.tw

Content

1. Details of constructing PS-zeolite systems 3
2. Styrene affinity and important features 4
3. Feature correlations 5
4. Comparison of main products observed in experiments and simulations 7
5. Pyrolysis of pure PS 8
6. Light species for PS-ASV system 10
7. Comparison in loading capacity between GCMC and MD 11
8. Comparison between loading capacity and Henry's constant 14
9. Computational details of the relative density distribution 15
10. Relative density distribution and carbon atom disrtibution 16
References 17

1. Details of constructing PS-zeolite systems

Table S1 presents the zeolite name, the maximum diameter of a sphere that can diffuse along x, y, and z directions (denoted as maxdsd_a (Å), maxdsd_b (Å), and maxdsd_c (Å), respectively), the dimensions of the unit cell along the x, y, and z directions (denoted as a (Å), b (Å), and c (Å), respectively), and the number of repeated units adopted in the x, y, and z directions (denoted as n_a, n_b, and n_c, respectively). All the dimensions are given in angstroms (Å). The zeolite structures listed include ASV(001), AWW(001), ETR(001), FER(001), MTF(001), PCR(001), PWW(001), SZR(001), UOS(010), MFI(001), MFI(010), MFI(100), AEL(001), and UOZ(100). The surface is cleaved according to the maxdsd; for example, the (001) surface is cleaved from ASV since only along the z direction can allow the diffusion of styrene molecules. The number of repeated units is chosen to make the size of the zeolite part to be close to 50 Å × 50 Å × 30 Å.

Table S1: Details of constructing zeolite structures in the PS-zeolite pyrolysis systems

Zeolite name	maxdsd_a (Å)	maxdsd_b (Å)	maxdsd_c (Å)	a (Å)	b (Å)	c (Å)	n_a	n_b	n_c
**ASV(001)**	1.52	1.52	4.43	8.67	8.67	13.91	6	6	2
**AWW(001)**	1.86	1.86	4.17	13.63	13.63	7.62	4	4	4
**ETR(001)**	2.85	2.85	9.33	20.63	20.63	8.42	3	3	3
**FER(001)**	1.56	3.4	4.69	19.02	14.3	7.54	3	4	4
**MTF(001)**	1.58	1.49	4.03	9.63	30.39	7.25	5	2	4
**PCR(001)**	1.51	3.16	4.2	20.14	14.07	12.52	3	4	2
**PWW(001)**	1.55	2.91	4.85	20.76	11.71	11.04	3	4	3
**SZR(001)**	3.32	3.32	4.69	18.86	14.4	7.51	3	4	4
**UOS(010)**	2.6	4.24	3.05	19.9	7.55	9.06	3	4	6
**MFI(001)**	4.7	4.46	4.46	20.09	19.73	13.14	3	3	2
**MFI(010)**	4.7	4.46	4.46	20.09	19.73	13.14	3	2	4
**MFI(100)**	4.7	4.46	4.46	20.09	19.73	13.14	2	3	4
**AEL(001)**	4.63	2.01	2.01	8.31	18.72	13.39	6	3	2
**UOZ(100)**	1.49	1.49	1.32	8.62	8.62	27.54	4	6	2

2. Styrene affinity and important features

The log(S) is found to correlate with LCD; the highest log(S) is found with LCD values between 5 Å and 6 Å, but overall, in a weak manner, as shown in Figure S1a. This is reasonable given the value of LCD can only represent a local property of the structure. At the same time, the interactions between adsorbates and adsorbents are not considered. In Figure S1b, the log(S) of zeolites shows a positive correlation with the adsorption energy of AMS. The compactness of the pore space can be seen as an important feature in the difficulty of adsorption of AMS. As a result, structures with the correct geometric configuration have a greater capacity to separate styrene from AMS. That is, affinity is indeed controlled by energetic features, but it is also related to geometric features.

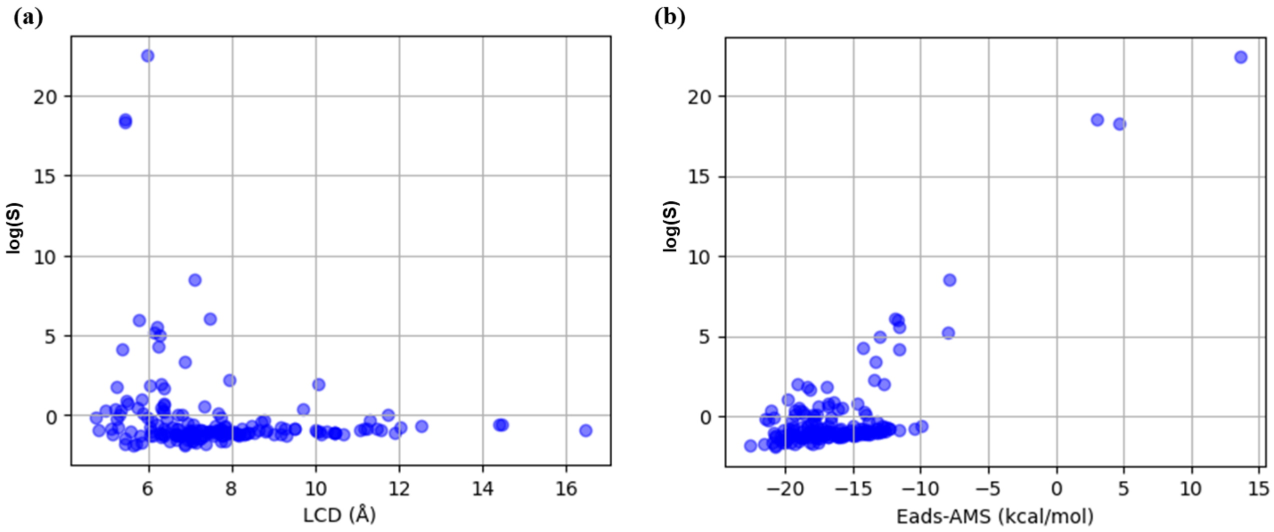

Figure S1: Relationships between the log(S) of candidates and their **(a)** LCD and **(b)** AMS adsorption enthalpy (Eads-AMS).

3. Feature correlations

The heatmap, shown in Figure S2, presents a comprehensive analysis of inter-property relationships in the context of styrene and AMS (Alpha-methylstyrene). The map employs a color-coded scheme to indicate the strength and direction of correlation coefficients between various physical and chemical descriptors. The maximum diameters of the largest sphere that can diffuse through the framework along different directions are labeled as Max dsd(a), Max dsd(b), and Max dsd(c). PLD (pore limiting diameter) and LCD (largest cavity diameter) are structural parameters that define pore sizes. Accessible area and accessible volume denote the surface area and volume within the zeolite accessible to the adsorbates. The largest ring represents the size of the largest ring structure within the zeolite framework. FDSi (framework density of silicon) indicates the silicon density within the zeolite framework. Adsorption energies, Eads-AMS and Eads-Styrene, are the interaction energies between the framework and the respective adsorbates AMS and Styrene. Besides, d1 represents the smallest Si-H Distance (H from Styrene, Si from zeolite framework), d2 represents the smallest O-H Distance (H from Styrene, O from zeolite framework), d3 represents the smallest Si-H Distance (H from AMS, Si from zeolite framework), d4 represents the smallest O-H Distance (H from AMS, O from zeolite framework). Notable correlations emerge between properties such as "Max dsd(c)" and "PLD," suggesting a strong positive linear relationship as indicated by the predominance of green hues. Conversely, "Largest ring" exhibits a significant negative correlation with "d4 ", underscored by a purple hue. Log(S) appears to display varying degrees of correlation with the descriptors analyzed, illustrated through the map where different shades are present, signifying the strength and nature of these correlations. Also, log(S) and the adsorption energy of AMS have a strong positive correlation (see the main text).

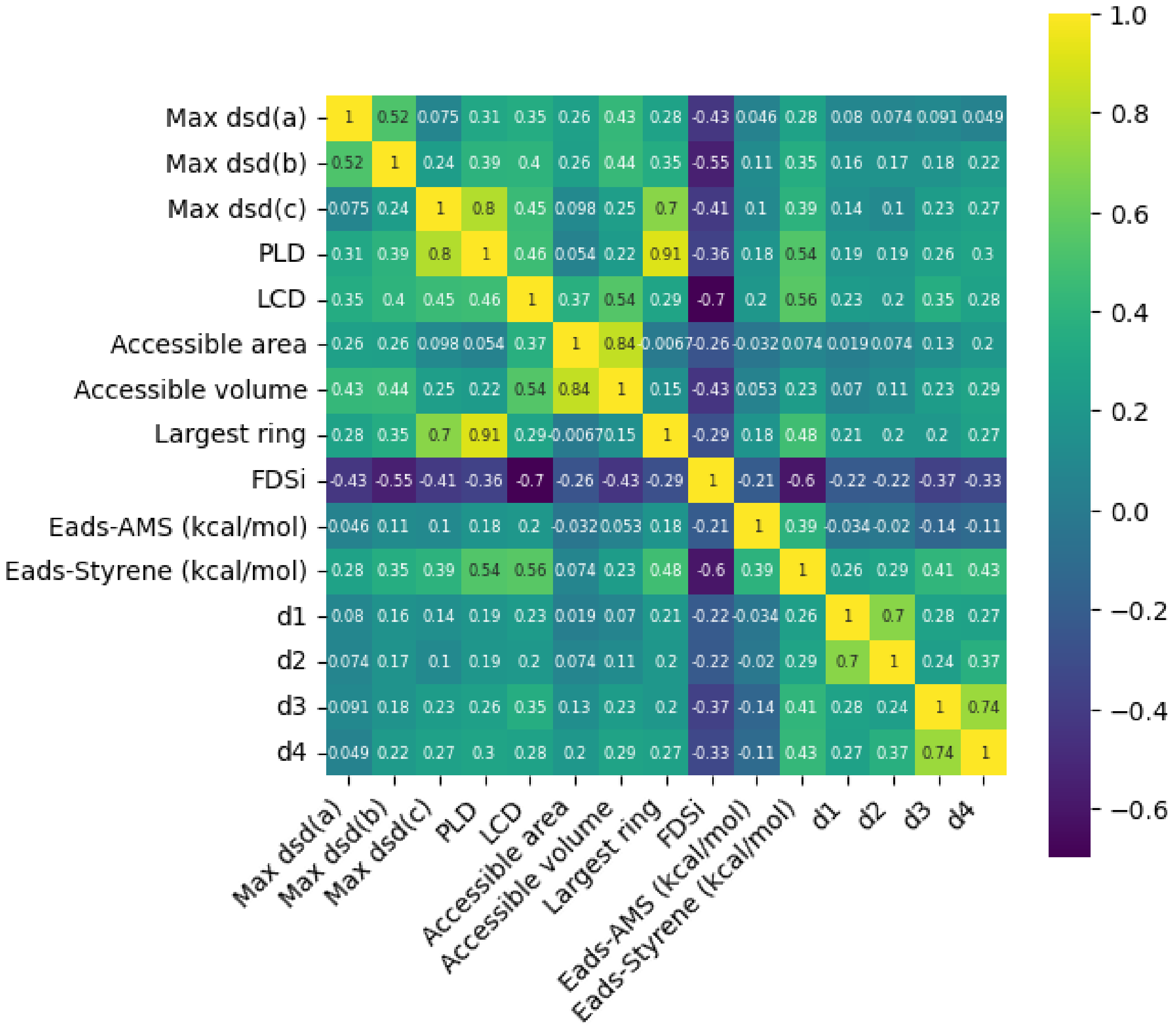

Figure S2: Heatmap for the correlations between features and adsorption properties. The corresponding Pearson correlation coefficients are color-coded. The warmer (colder) color indicates that the binary combination is more positively (inversely) correlated.

4. Comparison of main products observed in experiments and simulations

Table S2 lists the main products observed from previous experiments of PS pyrolysis and our simulations; MD results show good agreement with experimental observations[1], except xylene, which is not observed in MD since the formation of xylenes involves the fracture and reorganization of CH_3 and toluene fragments, which requires longer simulation time and higher temperatures. This is also reflected in the lack of xylenes in the early-stage products observed experimentally. As mentioned in the main text, the major products are low boiling fraction of styrene and α-methylstyrene and medium boiling fraction components, including 1,2-Diphenylethane, 1,3-Diphenylpropane, 2,4-Diphenyl-1-butene and 2,4-Diphenyl-1-pentene which is also consistent with experimental observation[2–4].

Table S2: Comparison of main products observed from experiments and simulations (1ns simulation under 1800 K) for PS pyrolysis.

Pyrolysis products of PS	Experiments	Simulation
Hydrogen	×	×
Ethylene	×	×
Ethane	×	√
Benzene	√	√
Toluene	√	√
Ethylbenzene	√	√
Xylene	√	×
Styrene	√	√
α-Methylstyrene	√	√
Stilbene	√	√
1,2-Diphenylethane	√	√
1,3-Diphenylpropane	√	√
2,4-Diphenyl-1-butene	√	√
2,4-Diphenyl-1-pentene	√	√
2,4,6-Triphenyl-1-hexene	√	√

5. Pyrolysis of pure PS

Reactive MD simulations are conducted to study pure PS pyrolysis, with a focus on understanding the major species and the reaction mechanism. These will also serve as a critical basis for further comparison with the zeolite-assisted PS pyrolysis. To first verify the accuracy of the simulations, Figure S3a shows the activation energy of the PS pyrolysis determined by MD simulations to be 313.40 kJ/mol, which is in good agreement with the value of 314.4 kJ/mol in previous experimental kinetic results.[5] This agreement suggests the accuracy and robustness of the ReaxFF method in studying the reactive behavior of pyrolysis systems. Aside from the activation energies, the observed products in simulations also generally resemble that of experimental results (Table S2), again validating the ReaxFF's capacity to capture the chemical composition of pyrolysis processes.

For the pure PS pyrolysis process, Figure S3b shows that the PS chain cracking is a fast process with the quick release of small molecules; at 1800 K, the number of carbon atoms in the longest chain reduced rapidly, as noted that the longest chain length becomes less than 100 within 340 ps, starting from an initial chain with 1600 carbon atoms. The chain length will not further increase after reaching the minimum point, indicating that short-chain species will no longer merge into longer-chain species for PS pyrolysis. To further understand pure PS pyrolysis, the evolution of species within a simulation time of one ns is studied. All species observed in simulations are classified into six categories based on their chain length (i.e., denoted as n−m, representing the range in the number of carbon atoms), as shown in Figure S3c. For simplicity and clarity, $C_{1600-100}$ and C_{99-31} represent all long and medium chains. In contrast, C_{30-21}, C_{20-11}, C_{10-5}, and C_{4-1} respectively represent the trimer species (2,4,6-triphenyl-1-hexene and its derivatives), dimer species (2,4-diphenyl-1-butene and its derivatives), monomer species (such as styrene and α-methyl styrene), and light gas species (such as ethylene, acetylene). The long- and mid-chain species are generated and consumed from the breaking process, showing a peak. The main gas species with a carbon number from 21 to 30 are generated at the beginning of the reaction, but overall, the number is lower than 10. The C_{20-11} species have similar behavior as $C_{30-21.}$ Still, with a number of 30, which is significantly higher than those longer-chain species, there is no significant further

consumption after those species are generated.

Figure S3d indicates that as the major species of PS pyrolysis, C_8 species are released rapidly, and Figure S3e shows that light species (with a carbon number lower than 6) can be formed in the beginning stage of the reaction along with the production of styrene. Figure S3f shows that the most prominent species during the reaction, which increase to relatively high levels compared to others, are stilbene, styrene, α-methylstyrene, and ethylene. Besides, it is also noted that ethylene is not directly related to the decomposition of styrene or α-methylstyrene since the C_7 species are at a low number and the C_8 species are majorly styrene. Instead, ethylene molecules are produced by a reaction, in which stilbene is involved; the details of the reaction mechanism will be discussed in the reaction mechanism section.

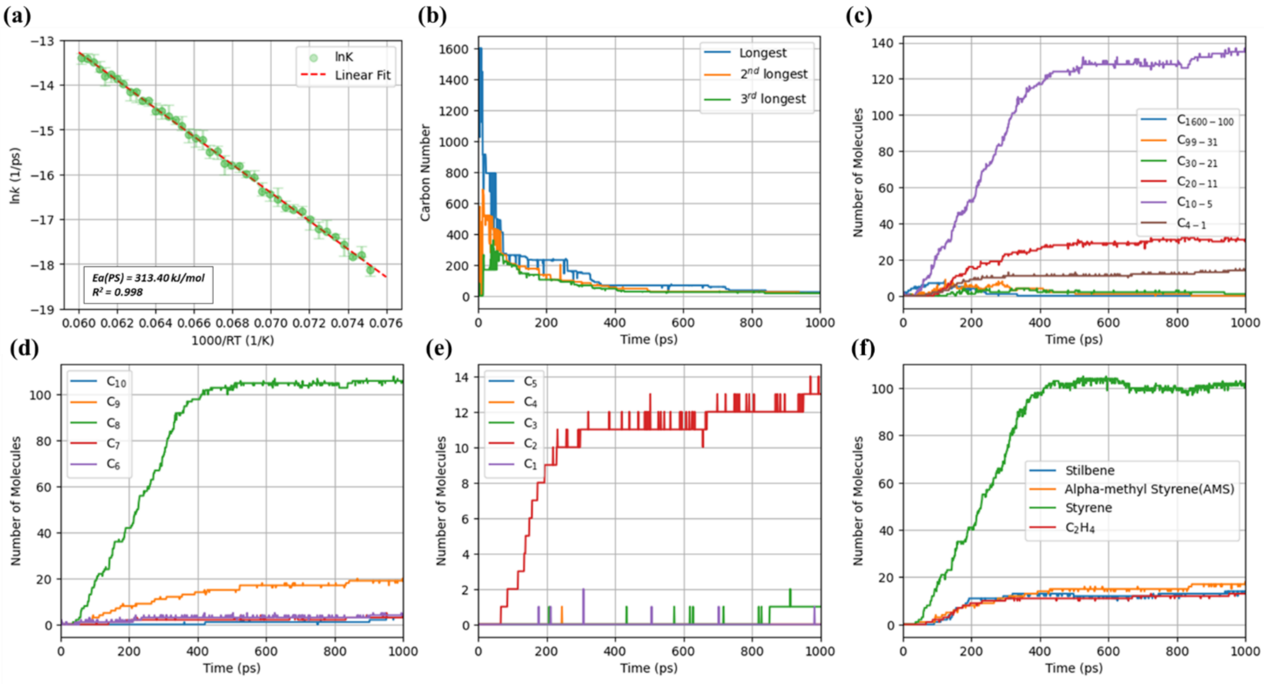

Figure S3: **(a)** MD-determined activation energy (E_a) for PS pyrolysis. The logarithm of reaction rate constants calculated from the mass loss ratio (i.e. $(m_{total} - m_{solid})/m_{total}$) is plotted versus 1000/T (T stands for temperature). **(b)** Time evolution of the carbon number of 1st, 2nd, and 3rd longest chain species observed at 1800 K. **(c)** Time evolution of species classified with carbon numbers at 1800 K. **(d)** Time evolution of species with carbon numbers from 6 to 10 at 1800 K. **(e)** Time evolution of species with carbon numbers from 1 to 5 at 1800 K. **(f)** Time evolution of major species at 1800 K.

6. Light species for PS-ASV system

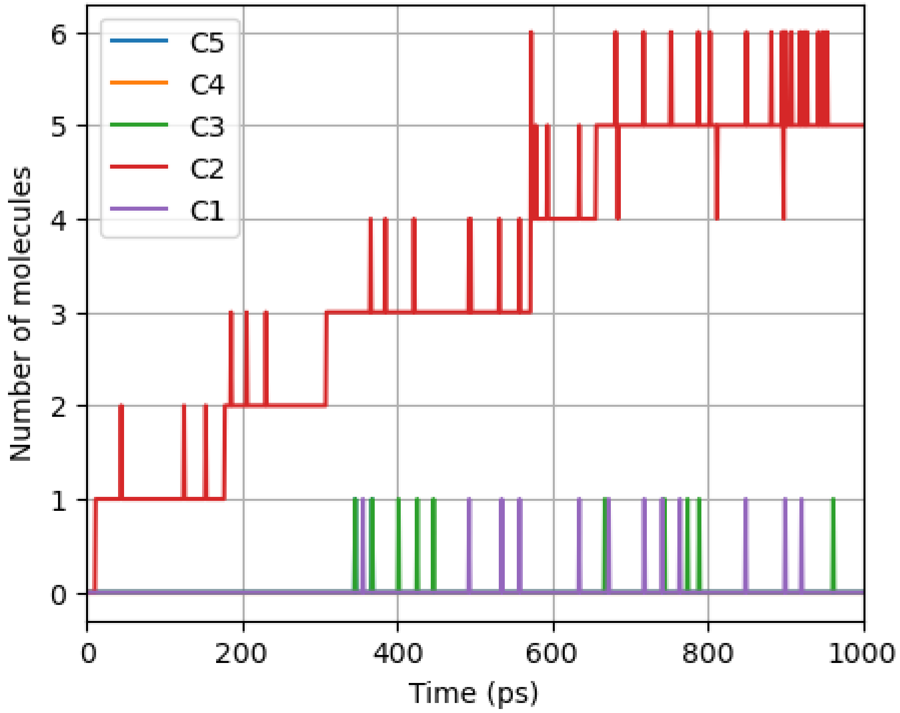

Figure S4: Time evolution of species with carbon numbers from 1 to 5 at 1800 K for PS-ASV.

Figure S4 illustrates the time evolution of species with carbon numbers ranging from 1 to 5 at 1800 K during the zeolite-assisted PS pyrolysis (PS-ASV system). The graph shows that C_2 species (in red) dominate the product distribution (compared with other species), with their number increasing progressively over time. This trend suggests that C_2 species are primarily generated through chain-breaking and olefin metathesis reactions, which are prominent pathways under the high-temperature conditions employed in this simulation. In contrast, species with carbon numbers C_1, C_3, C_4, and C_5 appear intermittently and in much lower quantities, indicating that the formation of these species is less favored or occurs only as minor by-products. The periodic generation of C_1 (purple) and C_3 (green) species suggests possible transient intermediates or secondary reactions leading to their appearance. Overall, this figure provides insight into the preferential formation of smaller hydrocarbons during PS pyrolysis in the presence of zeolite ASV, underscoring the impact of the zeolite interface on product distribution.

7. Comparison in loading capacity between GCMC and MD

Figure S5 presents a comprehensive study of styrene and AMS adsorption across a variety of zeolite materials. Figure S5a reveals distinct adsorption behaviors from GCMC simulations, particularly at (near) saturation region. In Figure S5b, ReaxFF simulations show styrene loadings between 0.2 and 0.3 mol/kg for different zeolites; compared to the GCMC outcomes, it is evident that saturation has not been achieved for these zeolites. Despite so, there is already a noticeable deviation from the linear regime (or the Henry's region), except for UOZ with zero loading. This explains the finding shown in the main text that the styrene loading positively correlates with $KH_{styrene}$ but does not increase with the $KH_{styrene}$ at the same order and magnitude. The loading of AMS demonstrates similar deviations from Henry's region as styrene but with some differences: Figures S5c and S5d illustrate that for AMS adsorption, AWW, SZR, and ASV have reached saturation when comparing ReaxFF and GCMC loadings while other systems deviate from Henry's region. In the ReaxFF MD simulations, the production of AMS from PS cracking is inherently limited, which in turn restricts the maximum loading capacity for zeolites, regardless of their affinity for AMS. This means that even in zeolites with a high AMS affinity, such as AEL, the low quantity of AMS produced from PS cracking prevents these zeolites from achieving high AMS loading capacities. In this study, we note that all zeolite structures are considered rigid in the GCMC simulations, while in reactive MD simulations structures are fully flexible. The framework flexibility could potentially affect loading capacity, but according to a previous study[6], tests conducted on a series of adsorbates have demonstrated that the impact of zeolite framework flexibility on adsorption is generally minimal.

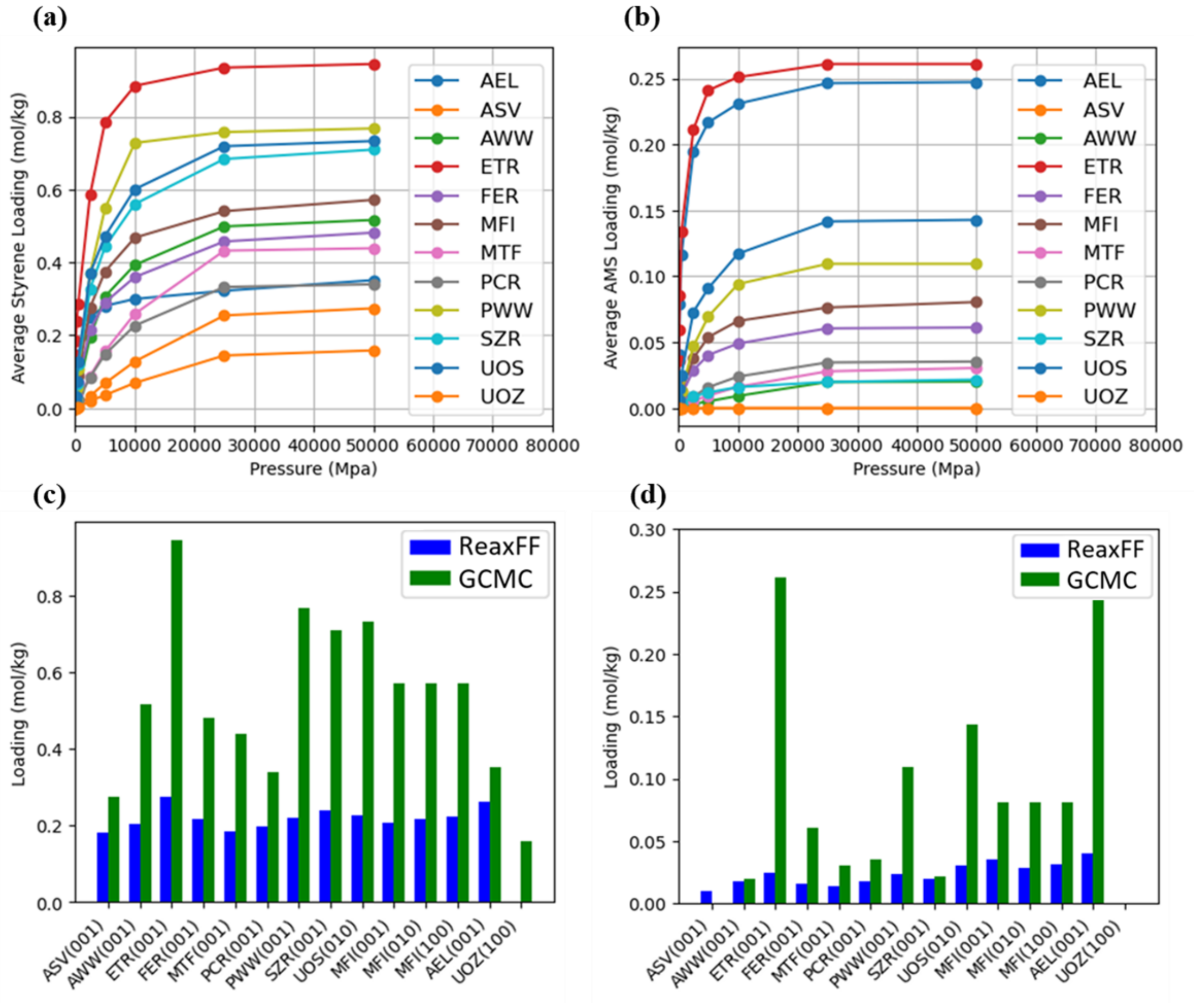

Figure S5: The GCMC-computed adsorption isotherms of **(a)** styrene and **(b)** AMS. The saturation loading from GCMG under 1800 K and the loading in zeolites from ReaxFF MD of **(c)** styrene and **(d)** AMS.

It is noted that in ASV, the AMS loading from ReaxFF MD is beyond the GCMC results (and ASV is the only zeolite that shows such a trend). To further study the difference of loading. Figures S6a and S6b illustrate the temporal dynamics of the center of mass for styrene and AMS within the PS-ASV and PS-UOZ systems over one nanosecond at 1800 K. Styrene, represented by grey dots, exhibits different behavior compared to AMS, denoted by red dots. In Figure S6, the presence of styrene molecules in the zeolite channels is interestingly found to block AMS diffusion, leading to higher AMS loading despite low AMS affinity, which leads to the observation that even in AMS-phobic zeolites such as ASV, AMS can still be accommodated. This happens because those styrene molecules enter the zeolite after AMS can subsequently block AMS within the zeolite. This blocking effect allows AMS to remain in the zeolite despite its initial aversion, resulting in a higher-than-predicted loading of AMS during ReaxFF MD simulation when compared with GCMC simulation (as shown in Figure S5d). As for the PS-UOZ system, the limitation of PLD prevents both the styrene and AMS molecules from entering the zeolite and leads to zero loading during the ReaxFF MD simulation. These findings further underscore the significant impact of zeolite structures on adsorption behavior and further explain the difference in loading in the ReaxFF MD and GCMC simulations.

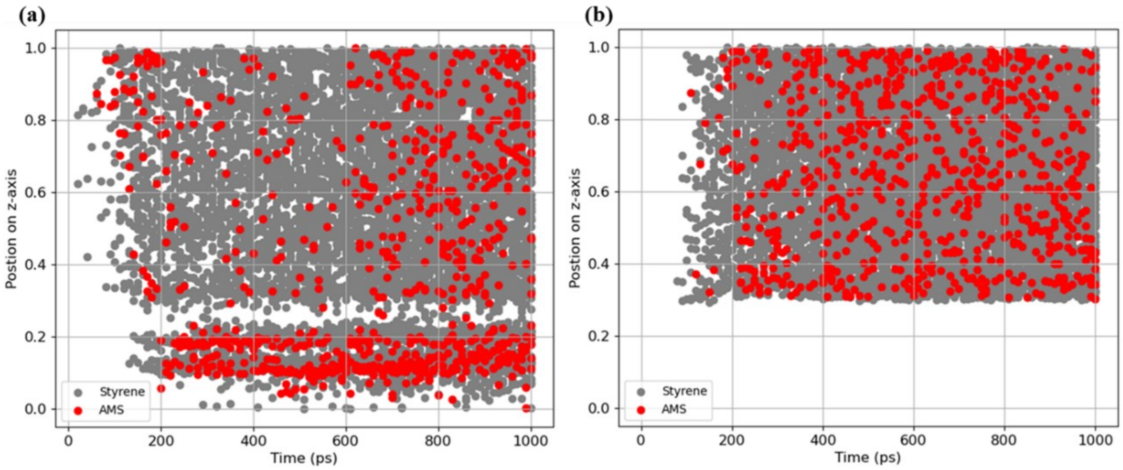

Figure S6. Time evolution of the center of mass for styrene (grey dot) and AMS (red dot) in **(a)** PS-ASV system and **(b)** PS-UOZ system from 0-1 ns at 1800K.

8. Comparison between loading capacity and Henry's constant

Figure S7 illustrates the alignment of selectivity calculated from the ReaxFF MD simulation with the S (S = $KH_{Styrene}/KH_{AMS}$). It is observed that selectivity can reach near-zero values, aligning with predictions from Henry's constant. However, AMS-phobic zeolites such as ASV, AWW, and SZR demonstrate higher selectivity of AMS than the prediction of S. This discrepancy in selectivity, where AMS selectivity calculated from ReaxFF MD simulations are higher than those calculated from KH, is likely due to the dynamic interactions of adsorbates within the zeolites. Specifically, styrene molecules entering the zeolites after AMS may obstruct the exit of AMS early in the reaction, compounded by high KH values that restrict styrene mobility within the zeolite matrix, enhancing this effect. Conversely, AMS-favorable zeolites like AEL, which have high KH_{AMS}, exhibit lower selectivity than other zeolites. This is because the limited amount of AMS produced during the early stages of pyrolysis does not reach the KH ratio, while the abundance of styrene allows it to enter even less styrene-preferred zeolites. Despite these dynamics, the proportional relationship between KH and loading is maintained to some extent, validating the use of KH as a descriptor for selecting zeolites that optimize styrene loading.

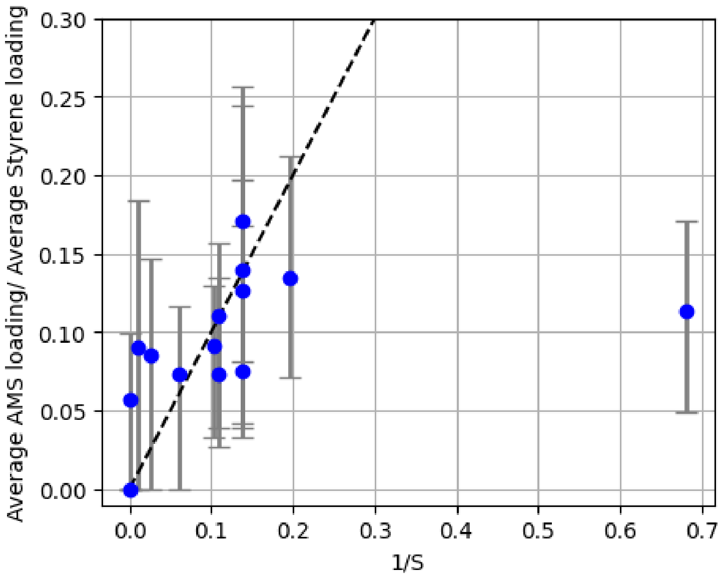

Figure S7: Comparison of selectivity from ReaxFF MD Simulations and Henry's Constant Calculations. The graph plots the average AMS and styrene loading against the inverse selectivity ratio (1/S). The blue dots represent the average loading values observed in ReaxFF simulations, while the dashed line indicates the expected trend based on Henry's constant calculations.

9. Computational details of the relative density distribution

To analyze the density distribution of the PS polymer, several key equations are utilized. The center of mass coordinates (x_{cm}, y_{cm}, z_{cm}) of the system are determined using Equation (S1), which calculates the mass-weighted average position of all carbon atoms in the system. Here, N represents the total number of carbon atoms, m_c is the mass of the carbon atom, and x_i, y_i, and z_i are the coordinates of the i-th carbon atom, with M being the total mass of carbon atoms in the system. Equation (S2) is employed to calculate the Euclidean distance R from the center of mass to a specific point (x_c, y_c, z_c) in space. This measure is crucial for understanding how far certain particles or regions are from the system's center of mass, providing insight into the spatial distribution within the PS polymer. To compare the density of carbon atoms within a specific radial distance R in the PS with zeolite system to that in the pure PS system, Equation (S3) is used. This normalized density within the range of R ($\rho_N(R)$) is defined as the ratio of the number of carbon atoms within distance R in the PS with zeolite system to that in the pure PS system. This comparison highlights the influence of zeolite on the density distribution of carbon atoms, indicating how zeolite's presence can alter the structural arrangement of the polymer.

$$x_{cm} = \frac{\sum_{i=1}^{N} m_c x_i}{M} \quad y_{cm} = \frac{\sum_{i=1}^{N} m_c y_i}{M} \quad z_{cm} = \frac{\sum_{i=1}^{N} m_c z_i}{M} \tag{S1}$$

$$R = \sqrt[2]{(x_c - x_{cm})^2 + (y_c - y_{cm})^2 + (z_c - z_{cm})^2} \tag{S2}$$

$$\rho_N(R) = \frac{\sum_{d<R} N(Carbon)_{PS\ with\ Zeolite}}{\sum_{d<R} N(Carbon)_{PS}} \tag{S3}$$

10. Relative density distribution and carbon atom disrtibution

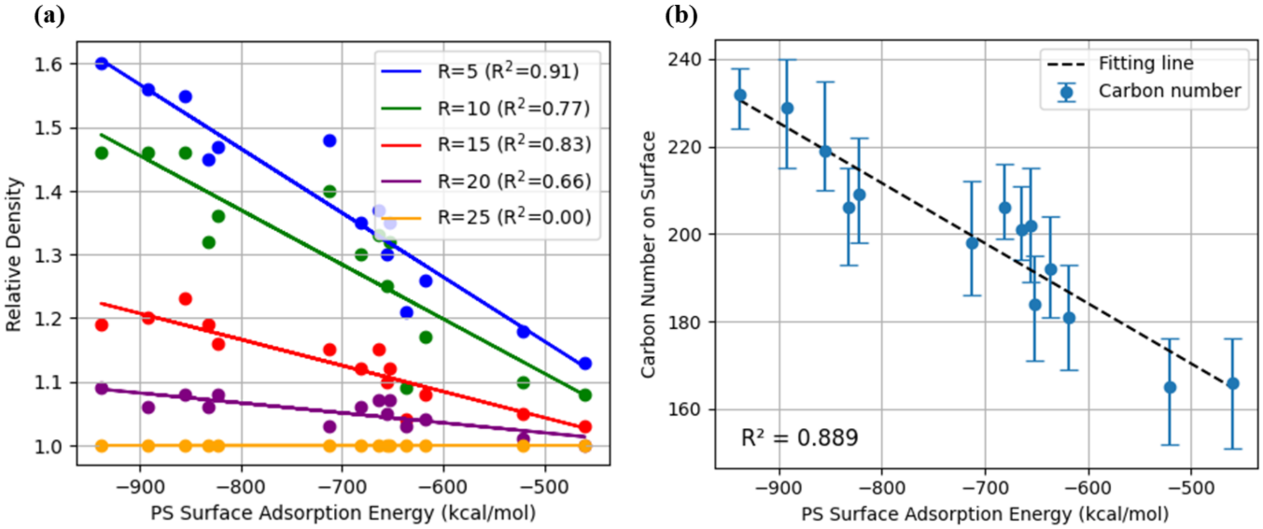

Figure S8. **(a)** Relationship between the relative density and surface adsorption energy. **(b)** Carbon number near zeolite interface vs. PS surface adsorption energy.

Figure S8 examines the relationship between PS surface adsorption energy and two indicators of polymer configuration near the zeolite surface: relative density and carbon number. Figure S8a shows the relative density of PS as a function of surface adsorption energy at various distances (R values) from the surface. A clear trend is observed, with higher relative densities corresponding to stronger (more negative) adsorption energies, especially closer to the surface (R=5, shown in blue, with an R^2 of 0.91). This trend diminishes with increasing distance, indicating that the influence of surface adsorption energy is localized primarily near the interface. Figure S8b presents the relationship between the carbon number of PS segments near the zeolite interface and PS surface adsorption energy. The negative correlation ($R^2 = 0.889$) suggests that as surface adsorption energy becomes stronger, fewer carbon atoms are present on the surface, reflecting a more compact polymer configuration. These findings highlight the significant role of surface adsorption energy in modulating the density and spatial arrangement of PS at the zeolite interface, potentially influencing the pyrolysis process and product distribution.

References

(1) Liu, Y.; Qian, J.; Wang, J. Pyrolysis of Polystyrene Waste in a Fluidized-Bed Reactor to Obtain Styrene Monomer and Gasoline Fraction. *Fuel Processing Technology* **2000**, *63* (1), 45–55. https://doi.org/10.1016/S0378-3820(99)00066-1.

(2) Maafa, I. M. Pyrolysis of Polystyrene Waste: A Review. *Polymers* **2021**, *13* (2), 225. https://doi.org/10.3390/polym13020225.

(3) Cao, Y.; Chu, Y.; Wang, Z.; Qi, J.; Zhou, L.; Li, Z. Thermophysical Properties of Low-Density Polystyrene under Extreme Conditions Using ReaxFF Molecular Dynamics. *Molecular Physics* **2021**, *119* (8), e1878304. https://doi.org/10.1080/00268976.2021.1878304.

(4) Li, D.; Lei, S.; Wang, P.; Zhong, L.; Ma, W.; Chen, G. Study on the Pyrolysis Behaviors of Mixed Waste Plastics. *Renewable Energy* **2021**, *173*, 662–674. https://doi.org/10.1016/j.renene.2021.04.035.

(5) Navarro, M. V.; López, J. M.; Veses, A.; Callén, M. S.; García, T. Kinetic Study for the Co-Pyrolysis of Lignocellulosic Biomass and Plastics Using the Distributed Activation Energy Model. *Energy* **2018**, *165*, 731–742. https://doi.org/10.1016/j.energy.2018.09.133.

(6) Daou, A. S. S.; Findley, J. M.; Fang, H.; Boulfelfel, S. E.; Ravikovitch, P. I.; Sholl, D. S. Quantifying Impact of Intrinsic Flexibility on Molecular Adsorption in Zeolites. *J. Phys. Chem. C* **2021**, *125* (9), 5296–5305. https://doi.org/10.1021/acs.jpcc.0c09952.
